# First-year university is associated with greater body weight, body composition and adverse dietary changes in males than females

**DOI:** 10.1371/journal.pone.0218554

**Published:** 2019-07-03

**Authors:** Kayleigh M. Beaudry, Izabella A. Ludwa, Aysha M. Thomas, Wendy E. Ward, Bareket Falk, Andrea R. Josse

**Affiliations:** 1 Department of Kinesiology, Faculty of Applied Health Sciences, Brock University, St. Catharines, Ontario, Canada; 2 Centre for Bone and Muscle Health, Faculty of Applied Health Sciences, Brock University, St. Catharines, Ontario, Canada; 3 School of Kinesiology and Health Science, Faculty of Health, York University, Toronto, Ontario, Canada; McMaster University, CANADA

## Abstract

**Background:**

The transition from high school to university life is a critical time for change, often accompanied by the adoption of negative lifestyle habits including unhealthy nutrition. The purpose of this longitudinal study was to identify sex-specific changes in dietary intake and diet quality, and associated changes in body weight and composition during first-year university.

**Methods:**

Three-hundred and one students (n = 229 females) completed food frequency questionnaires, and had their body weight, body composition, waist and hip circumference measured at the beginning and end of first-year university. Repeated-measures ANOVAs with covariate adjustments were used with variables for sex (between group) and time (within group) to assess these changes.

**Results:**

Students gained body weight and fat during the year (p<0.001). Body mass Index (BMI) also significantly increased (p = 0.032). Males gained more weight (Male:3.8 kg; Female:1.8 kg), fat mass (Male:2.7 kg; Female:1.5 kg), lean mass (Male:1.1 kg; Female:0.3 kg) and BMI (Male:1.2 kg/m^2^; Female:0.7 kg/m^2^; p≤0.001 for interactions), and had greater increases in waist circumference (Male:2.7 cm; Female:1.1 cm) and waist:hip ratio (Male:0.02; Female:0.004; p<0.05 for interactions) than females. Energy intake remained the same over the year in both sexes, accompanied by an increase in alcohol (ethanol) in both sexes but more so in males than females (p = 0.011 interaction). Diet quality decreased, characterized by a reduced intake of healthy foods/beverages (p<0.05) in both sexes such as yogurt, cheese, oatmeal, breads, rice, pasta, vegetables, green salad, fruits, steak, fish, nuts and milk, and an increased consumption of unhealthy foods and beverages (p<0.05) such as donuts/cakes, fried chicken, beer and liquor. Significant interactions between sexes indicated that males displayed a more adverse and lower quality eating pattern which included greater intakes of donuts/cakes, fried chicken, beer and liquor, as well as decreased intakes of eggs and vegetables compared to females. Lastly, some dietary intake changes significantly correlated with fat mass and waist circumference change indicating that poor dietary choices were associated with increased adiposity.

**Conclusions:**

Our study demonstrated that during first-year university, both male and female students undergo unfavorable changes in nutrition and body weight/composition that significantly differ between sexes, with males showing more adverse changes. Our results can be used to inform effective sex-specific strategies and interventions to improve dietary habits during the transition to university life.

## Introduction

The worldwide rates of overweight and obesity are increasing [1]. According to a 2015 report, approximately 604 million adults or 12% of the population were considered obese worldwide, with North America having the highest prevalence of obesity [2]. When looking at patterns of body weight change over the lifespan, a critical time for weight gain appears to be in later adolescence and early adulthood [3–5]. During adolescence, patterns of sex-specific weight gain also emerge such that males tend to gain more lean mass while females gain more fat mass [6]. This time coincides with the transition from secondary school to university or college. During this time, individuals are beginning to settle into their life routines and are adopting lifestyle habits that are likely to be sustained into later adulthood [7, 8]. These lifestyle habits may lead to weight and fat gain which could have negative long-term health implications [7–12]. In most [3, 5, 13–15] but not all [16] studies, males have been shown to gain more body weight than females. Little is known about the composition of the weight gained [15], and studies directly comparing this in males versus females are scarce. Nevertheless, adolescent and young adult weight gain is highly linked to overweight and obesity in later adulthood [17]. Thus, during critical time periods, like the transition from high school to university life, it is important to identify, characterize and assess the impact of certain determinants that may contribute to obesity and negatively impact long-term overall health and wellbeing in males and females [18, 19]. This may help combat the rising rates of adult obesity and associated co-morbidities [7, 20, 21].

The etiology of obesity is multifactorial [22], and there are several features which influence the habits of students in first-year university that can lead to weight gain and negative body composition change [23, 24]. One of these is dietary intake. Excessive consumption of calorie-dense foods, poor meal consumption patterns, increased dieting, and disordered eating have all been associated with undergraduate weight gain [25–27]. However, nutrition-related findings, particularly in North America, are conflicting [28–31]. For example, two Canadian studies found no significant change in energy and nutrient intake over the course of first-year university in males [28] and females [30], while two studies in female first-year students reported significant decreases in nutritional intake, despite increases in body weight [29, 31].

Beyond energy and nutrient intake, it is also important to investigate changes in dietary patterns and diet quality. Dietary patterns of healthy or unhealthy eating, rather than intakes of individual foods or nutrients, are more closely linked to chronic disease risk [32–34]. Diet quality is used to describe how well an individual’s diet conforms to dietary recommendations, thereby providing appropriate amounts of all essential nutrients [35]. Diet quality in both males and females has been shown to decrease over the course of first-year university, and is characterized by a general decrease in the consumption of fruits, vegetables and whole grains, and an increase in the consumption of foods containing high levels of sodium, sugar and saturated fat [4, 24, 36, 37]. There have also been instances of increased alcohol intake over the course of first-year university in both male and female students which is also indicative of a decrease in overall diet quality [24, 29, 37, 38]. These negative dietary changes are associated with adverse health consequences [12]. However, none of these studies assessed changes in nutrient intake and diet quality along with changes in body composition, and none explored these changes between the sexes. Therefore, the objective of our study was to examine sex-specific changes in body weight, body composition, waist/hip circumference, dietary intake and diet quality during first-year university. Our *a priori* hypotheses were: a) students will experience negative physical changes including increases in body weight and fat mass. Sex-specific differences in patterns of weight gain and body composition change will emerge; b) dietary intake will change and diet quality will decrease throughout the academic year characterized by a reduced consumption of healthy foods and beverages and an increased consumption of unhealthy foods and beverages; c) the change in healthy and unhealthy food consumption will differ between males and females; and d) adverse changes in nutrition will be associated with negative sex-specific physical changes.

## Materials and methods

### Study design and participants

A sample of incoming first-year male and female students were recruited though posters, flyers, information booths at university welcome week events, the media and word of mouth. Eligibility criteria included: age 17–20 years; attendance at Brock University (St. Catharines, Ontario, Canada), and no previous college or university experience. Written informed consent was obtained directly from the participants. The study was cleared by Brock University’s Bioscience Research Ethics Board (REB #13–297).

### Data collection

Two cohorts of participants (2014–2015 and 2015–2016) were followed longitudinally with data collected at 2 study visits: the beginning of their first-year (August/September) and the end of their first-year (March/April). Data collected included anthropometrics (height, weight, waist and hip circumference), body composition and dietary intake.

#### Anthropometrics and body composition

Body weight, body fat percent, lean body mass and fat mass were assessed using bioelectric impedance analysis (BIA; InBody520 bioelectrical impedance analysis system; Biospace Co. Inc. Los Angeles, CA, USA) with no shoes and light clothing. Participants were asked to arrive at the laboratory during the morning hours (0800–1100) after an overnight fast, and to refrain from exercise or alcohol consumption for 24 hours and 12 hours, respectively, prior to body composition analysis. Care was taken to minimize the effects of hydration status on our measures by having participants consume 500 ml of water upon arrival to the laboratory. After 30 min, they were asked to void and then BIA measurements were taken. Height was measured with a stadiometer to the nearest 0.1 cm with no shoes and light clothing, and body mass index (BMI) was calculated. Waist and hip circumference (cm) were measured using a standard, retractable, non-metallic tape measure placed at the waist at the level of the umbilicus, and across the largest part of the buttocks and below the iliac crest, respectively. Anthropometric measurements were done in duplicate and then averaged. Waist to hip ratio was calculated. The same researcher carried out the same measurements on the same subjects at the beginning and end of the study.

#### Dietary intake and diet quality

Dietary intake and diet quality were assessed using a food frequency questionnaire (FFQ; Block 2014, NutritionQuest, Berkeley, CA, USA). The FFQ included 127 commonly consumed food and beverage items, and assessed intakes retrospectively over 6 months [39]. FFQs were administered immediately upon starting university in August/September (results of which would reflect, as best as possible, intakes prior to entering university), and again, just before finishing first year in March/April (which would reflect intakes during first year). All data from the FFQs were analyzed by NutritionQuest using a Canadian nutrient database. For the diet quality assessment, dichotomous (i.e. healthy vs. unhealthy) categories of foods/beverages were created based on Canada’s Food Guide [40], the United States and Canada Healthy Eating Index [41, 42] and meta-analyses that assessed specific dietary factors/foods and chronic disease risk [43, 44].

#### Statistical analysis

All data were stratified by sex before analysis. For the body composition/anthropometry analyses, at each timepoint, outlying data points were identified and individually checked to determine if they were true outliers (i.e. participants who were tall, large etc., compared to the general distribution of our data) or outliers due to a data entry or measurement error. Data errors were subsequently corrected, and all other outliers were left in the dataset. For the nutrient and diet analyses, at each timepoint, female data were removed if total energy intake was calculated to be <500 kcals or >3500 kcals, (as was done in [45]). Similarly, male data were removed if energy intake was <1000 kcals and >4000 kcals. Differences in anthropometrics, body composition and dietary intake from the beginning and end of first-year university were determined using a two-way repeated measures analysis of variance (RMANOVA) with sex being the between-subject variable and time being the within-subject variable. Partial eta-squared values were calculated from the RMANOVA as a measure of effect size, where ~0.02 is considered a small effect, ~0.13 is a medium effect and ~ 0.26 is a large effect [46]. Several covariates that may have affected our outcomes were assessed separately in each RMANOVA model. These included: 3 levels of physical activity (light, moderate and vigorous; data reported elsewhere [47]) cohort (to account for potential clustering bias), faculty of study (Health Sciences, Arts, Business or Math/Science), and living arrangement (in residence, living with friends off campus or living at home). Covariates were retained in the models if their interaction with a main effect was significant, otherwise reduced models were presented. Plausible associations between dietary and body composition variables and between select dietary variables were determined by assessing 2-tailed Pearson correlations in males and females separately as well as in subgroups of those that gained body weight within each sex. Diet quality was assessed qualitatively by noting significant differences in intakes of foods (expressed as g/d) grouped into categories of healthy or unhealthy, over time and between sexes. Decreases in healthy foods and increases in unhealthy foods indicate an overall pattern of decreased diet quality. In addition, differences in intakes of these foods/beverages between sexes reflect differential changes in diet quality and eating patterns throughout the year. Statistical analyses were performed using SPSS (version 25, Chicago, Illinois, USA). P≤0.05 were considered statistically significant and those at 0.05<p <0.10 were reported as trends. We did not correct for multiple testing as done in [48].

## Results

### Study participants

A total of 1231 incoming first year students (380 males and 851 females) expressed interest in the study. Of those, 364 students completed the first study visit in September. 301 first-year students returned to complete the second study visit in March (83% retention rate). Reasons for dropping out were unknown but may relate to the fact that follow-up assessments were conducted around final exams (in March/April).

### Participant characteristics

Of the 301 participants, 24% were male (*n* = 72) and 76% were female (*n* = 229). Seventy two percent of students lived in residence (*n* = 218), 22% of students lived at home (*n* = 65) and 6% lived in a student house off campus (*n* = 17). Forty five percent of students were in the faculty of Applied Health Sciences (*n* = 136), 10% were in the faculty of Business (*n* = 29), 12% were in the faculty of Mathematics and Science (*n* = 36), 14% were in Education (*n* = 41), 17% were in Social Sciences (*n* = 50) and 3% were in Humanities (*n* = 9). Seventy four percent of the sample were Caucasian (*n* = 224), 15% were Asian (*n* = 44), 6% were African American (*n* = 17), 3% were Hispanic (*n* = 8) and the remaining 2% were of other ethnicities not stated above (*n* = 8).

### Anthropometrics and body composition

Table 1 displays the anthropometric and body composition results from the beginning and end of first-year university, separated and compared by sex. Both sexes gained body weight and fat mass and increased their waist:hip ratio. Males gained significantly more weight than females (interaction: p<0.001), increased their BMI, waist circumference and waist:hip ratio more than females (interactions: p = 0.001; 0.014; 0.046, respectively), gained more fat mass than females (interaction: p = 0.001), as well as more lean mass than females (interaction: p<0.001). In addition, the quality of weight gain was also sex-specific in that the weight gained by males was composed of 29% lean mass (71% fat), while the weight gained by females was 17% lean mass (83% fat). Height was unchanged. According to their mean BMI at the end of first-year (>25 kg/m^2^), males were now classified as ‘overweight’.

**Table 1:** Anthropometric and body composition data from beginning to end of first-year university in male (n = 72) and female (n = 229) students.

	Beginning	End	Change	Sex p and η^2^*	Time p and η^2^*	Interaction p and η^2^*
**Body weight (kg)**				<0.001; 0.26	<0.001; 0.36	<0.001; 0.075
Male	76.1 ± 12	79.9 ± 13	3.8 ± 4			
Female	61.4 ± 11	63.2 ± 12	1.8 ± 3			
**Height (cm)**				<0.001; 0.48	0.26; 0.004	0.85; <0.001
Male	178.4 ± 7	178.4 ± 7	0.0 ± 1			
Female	164.7 ± 6	164.7 ± 6	0.0 ± 1			
**Body Mass Index (kg/m**^**2**^**)**				0.005; 0.027	0.032; 0.015	0.001; 0.035
Male	23.9 ± 3	25.1 ± 4	1.2 ± 1			
Female	22.6 ± 4	23.3 ± 4	0.7 ± 1			
**Waist circumference (cm) **				0.001; 0.036	0.067; 0.011	0.014; 0.020
Male	81.5 ± 9	84.2 ± 9	2.7 ± 5			
Female	78.2 ± 10	79.3 ± 9	1.1 ± 5			
**Hip circumference (cm)†**				0.004; 0.028	0.002; 0.031	0.39; 0.002
Male	99.1 ± 7	100.6 ± 8	1.5 ± 5			
Female	96.2 ± 8	97.2 ± 9	1.0 ± 4			
**Waist to Hip Ratio**				0.023; 0.017	0.001; 0.037	0.046; 0.013
Male female	0.82 ± 0.06 0.81 ± 0.06	0.84 ± 0.05 0.82 ± 0.05	0.02 ± 0.05 0.004 ± 0.05			
**Fat Mass (kg)**				<0.001; 0.069	<0.001; 0.29	0.001; 0.036
Male	12.1 ± 6	14.8 ± 8	2.7 ± 3			
Female	17.5 ± 8	19.0 ± 8	1.5 ± 3			
**Body Fat (%)**				<0.001; 0.35	<0.001; 0.24	0.018; 0.019
Male	15.4 ± 6	17.8 ± 7	2.4 ± 3			
Female	27.6 ± 7	29.1 ± 7	1.5 ± 3			
**Lean Mass (kg)**				<0.001; 0.67	0.27; 0.004	<0.001; 0.035
Male	64.1 ± 8	65.2 ± 9	1.1 ± 2			
Female	43.9 ± 5	44.2 ± 5	0.3 ± 2			

All results are shown as mean ± SD

* Significance from 2-way RMANOVA (Group: sex; Time: beginning to end), significantly different with P value ≤0.05, and effect size was determined by partial eta-squared.

† Hip Circumference was not collected for one participant in each sex.

Body mass index with moderate PA and living arrangement as covariates; Waist and hip circumference with cohort as a covariate; Waist to hip ratio with vigorous PA as a covariate; Lean mass with faculty of study as a covariate.

### Dietary intake

Table 2 displays select nutrient intakes of both males and females from the beginning to end of first-year university. After covariate adjustment, total energy intake was not significantly different over the year or between sexes. Iron intake was significantly different between sexes (interaction: p = 0.009) and alcohol intake increased over the year in both sexes, but more so in males (interaction: p = 0.011). For all nutrients, males had greater intakes which was likely reflective of their greater total energy intakes.

**Table 2:** Nutrient intake data expressed as a unit per day from beginning to end of first-year university in male (n = 50) and female (n = 210) students.

Nutrient	Beginning	End	Sex p and η^2^*	Time p and η^2^*	Interaction p and η^2^*
**Energy (kcal)**			<0.001; 0.22	0.30; 0.004	0.55; 0.001
Male	2529 ± 797	2124 ± 665			
Female	1722 ± 627	1463 ± 521			
**Fat (g)**			<0.001; 0.19	0.48; 0.002	0.51; 0.002
Male	100.6 ± 33	84.4 ± 27			
Female	70.2 ± 28	58.4 ± 24			
**Saturated Fat (g)**			<0.001; 0.14	0.37; 0.003	0.29; 0.004
Male	33.2 ± 12	27.1 ± 10			
Female	23.1 ± 9	19.6 ± 9			
**Protein (g)**			<0.001; 0.26	0.99; <0.001	0.28; 0.005
Male	100.6 ± 35	81.1 ± 27			
Female	66.5 ± 25	52.7 ± 20			
**Carbohydrates (g)**			<0.001; 0.17	0.24; 0.005	0.38; 0.003
Male	302.4 ± 108	244.2 ± 92			
Female	217.6 ± 79	175.4 ± 65			
**Fibre (g)**			<0.001; 0.15	0.36; 0.003	0.21; 0.006
Male	24.5 ± 12	19.2 ± 12			
Female	16.8 ± 6	13.3 ± 6			
**Total Sugars (g)**			<0.001; 0.099	0.46; 0.002	0.11; 0.010
Male	140.0 ± 62	107.1 ± 47			
Female	100.9 ± 48	81.5 ± 37			
**Sodium (mg)**			<0.001; 0.19	0.91; <0.001	0.71; 0.001
Male	4029.4 ± 1339	3441.2 ± 1033			
Female	2868.8 ± 1089	2386.4 ± 934			
**Calcium (mg)**			<0.001; 0.11	0.81; <0.001	0.16; 0.008
Male	1309.9 ± 565	1025.3 ± 408			
Female	953.5 ± 402	769.1 ± 325			
**Iron (mg)**			<0.001; 0.30	0.47; 0.002	0.009; 0.026
Male	19.3 ± 7	15.0 ± 6			
Female	12.1 ± 4	9.6 ± 4			
**Alcohol (ethanol) (mg)**			0.001; 0.04	<0.001; 0.11	0.011; 0.025
Male	7.9 ± 12	13.8 ± 17			
Female	5.1 ± 9	7.3 ± 9			

All results are shown as mean ± SD

* Significance from 2-way RMANOVA (Group: sex; Time: beginning to end), significantly different with P value ≤0.05, and effect size was determined by partial eta-squared.

Energy, fat, saturated fat, protein, carbohydrate, fibre, sugars, sodium, calcium and iron with living arrangement as a covariate; Saturated fat with vigorous physical activity as a covariate; Iron with light physical activity as a covariate.

### Diet quality

We analyzed the FFQ data by assessing the amount of a food consumed per day, expressed as g/d. This measurement considers the frequency of consumption of a food (e.g. eating eggs 1 time per day or 1 to 3 times per week, etc.), as well as the amount consumed on each occasion (e.g. 2 eggs at one time). Tables 3 and 4 show these values for various foods that were designated as either part of a healthy diet (Table 3) or an unhealthy diet (Table 4). In Table 3, there were significant decreases in both sexes in the consumption of healthy foods including yogurt, cheese, oatmeal, breads, rice, pasta, vegetables, green salad, fruit, steak, fish, nuts and milk. Significant interactions were seen for eggs, vegetables (total) and starchy vegetables indicating that males decreased their intakes of these healthy foods more than females. The intakes of several healthy foods did not significantly change over time including eggs, chicken (not fried) and tea.

**Table 3:** Assessing diet quality: Foods that are generally seen as part of a *healthy* diet. Intakes (g/day) are from beginning to end of first-year university in male (n = 50) and female (n = 210) students.

Foods	Beginning	End	Sex p and η^2^*	Time p and η^2^*	Interaction p and η^2^*
**Eggs**			<0.001; 0.088	0.084; 0.012	0.036; 0.017
Male	34.4 ± 34	28.2 ± 34			
Female	15.1 ± 18	15.7 ± 21			
**Yogurt**			0.030; 0.018	0.050; 0.015	0.12; 0.010
Male	28.3 ± 43	15.9 ± 25			
Female	33.5 ± 37	32.1 ± 41			
**Cheese**			0.27; 0.006	0.002; 0.035	0.37; 0.003
Male	22.3 ± 23	18.8 ± 22			
Female	18.1 ± 20	16.2 ± 17			
**Oatmeal**			1.001; 0.040	0.007; 0.027	0.41; 0.003
Male	42.1 ± 67	31.0 ± 53			
Female	21.7 ± 33	15.7 ± 34			
**Breads** ^**a**^			<0.001; 0.064	<0.001; 0.12	0.11; 0.010
Male	59.9 ± 41	43.0 ± 30			
Female	40.5 ± 27	30.7 ± 23			
**Rice**			0.001; 0.045	0.031; 0.018	0.17; 0.007
Male	52.4 ± 50	49.0 ± 55			
Female	37.7 ± 54	22.0 ± 36			
**Pasta**			0.006; 0.029	0.002; 0.037	0.79; <0.001
Male	82.8 ± 66	73.7 ± 78			
Female	65.9 ± 76	49.7 ± 40			
**Vegetables** ^**b**^			0.003; 0.033	<0.001; 0.10	0.006; 0.029
Male	240.6 ± 239	172.1 ± 230			
Female	145.5 ± 110	119.3 ± 106			
**Green Salad**			0.008; 0.027	0.004; 0.032	0.70; 0.001
Male	43.9 ± 52	38.1 ± 63			
Female	28.5 ± 28	23.6 ± 28			
**Starchy Vegetables** ^**c**^			0.002; 0.037	<0.001; 0.13	0.005; 0.030
Male	454.1 ± 422	323.3 ± 404			
Female	285.5 ± 177	227.4 ± 178			
**Tropical Fruit** ^**d**^			0.034; 0.017	<0.001; 0.052	0.30; 0.004
Male	112.3 ± 111	80.8 ± 120			
Female	80.7 ± 69	62.8 ± 82			
**Temperate Fruits** ^**e**^			0.17; 0.007	<0.001; 0.048	0.83; <0.001
Male	64.1 ±73	50.1 ± 76			
Female	52.0 ± 48	37.6 ± 43			
**Steak**			0.002; 0.038	<0.001; 0.14	0.13; 0.009
Male	14.2 ± 15	7.1 ± 11			
Female	8.7 ± 11	4.0 ± 7			
**Chicken (not fried)**			0.030; 0.018	0.17; 0.007	0.86; <0.001
Male	33.8 ± 47	29.3 ± 34			
Female	23.1 ± 30	19.5 ± 31			
**Fish (Tuna/Salmon)**			0.012; 0.024	0.020; 0.021	0.44; 0.002
Male	13.0 ± 16	11.2 ± 24			
Female	9.3 ± 13	5.6 ± 10			
**Nuts**			0.079; 0.012	0.006; 0.029	0.62; 0.001
Male	14.7 ± 15	9.3 ± 12			
Female	10.6 ± 15	6.0 ± 9			
**Milk**			0.031; 0.018	<0.001; 0.11	0.61; 0.001
Male	205.9 ± 265	126.1 ± 184			
Female	147.2 ± 184	80.5 ± 125			
**Coffee**			0.028; 0.019	0.018; 0.022	0.085; 0.011
Male	73.9 ± 173	94.0 ± 152			
Female	48.6 ± 98	47.2 ± 103			
**Tea**			0.003; 0.034	0.25; 0.005	0.57; 0.001
Male	39.3 ± 144	21.1 ± 35			
Female	92.9 ± 155	86.7 ± 144			

All results are shown as mean ± SD

* Significance from 2-way RMANOVA (Group: sex; Time: beginning to end), significantly different with P value ≤0.05, and effect size was determined by partial eta-squared.

^a^ dinner rolls, white bread, multi grain bread, whole grain bread, whole wheat bread

^b^ all vegetables combined.

^c^ corn, potatoes, peas

^d^ melons, bananas, orange, tangerine, grapefruit, peach, nectarine, fruit salad

^e^ apples, pears, strawberries, other berries

Vegetables, green salad, starchy vegetables, steak, nuts with light physical activity as a covariate; nuts with moderate physical activity as a covariate; vegetables, starchy vegetables, nuts with vigorous physical activity as a covariate; coffee with living arrangement as a covariate; cheese, pasta, temperate fruit, chicken, nuts with faculty as a covariate.

**Table 4:** Assessing diet quality: Foods that are generally seen as part of an *unhealthy* diet. Intakes (g/day) are from beginning to end of first-year university in male (n = 50) and female (n = 210) students.

Foods	Beginning	End	Sex p and η^2^*	Time p and η^2^*	Interaction p and η^2^*
**Donuts/Cakes**			0.43; 0.002	<0.001; 0.059	0.035; 0.017
Male	9.4 ± 8	10.4 ± 14			
Female	10.2 ± 10	8.5 ± 11			
**Other Pastries** ^**a**^			0.057; 0.014	<0.001; 0.049	0.25; 0.005
Male	23.3 ± 28	16.4 ± 20			
Female	17.4 ± 15	13.9 ± 13			
**Pizza**			<0.001; 0.083	0.053; 0.014	0.84; <0.001
Male	29.5 ± 26	26.1 ± 23			
Female	17.2 ± 17	14.5 ± 17			
**Macaroni and Cheese**			0.54; 0.001	0.38; 0.003	0.78; <0.001
Male	15.4 ± 19	11.9 ± 18			
Female	12.2 ± 17	11.8 ± 27			
**Fried Chicken**			0.088; 0.011	0.004; 0.032	0.043; 0.016
Male	11.9 ± 14	20.8 ± 28			
Female	11.6 ± 15	13.2 ± 20.3			
**Fried Fish**			0.44; 0.002	0.006; 0.029	0.57; 0.001
Male	3.7 ± 6.2	2.0 ± 3			
Female	3.0 ± 6	1.8 ± 3			
**Meat Dishes** ^**b**^			<0.001; 0.15	<0.001; 0.068	0.85; <0.001
Male	91.0 ± 56	73.4 ± 60			
Female	55.1 ± 40	38.9 ± 31			
**French Fries**			0.19; 0.007	0.091; 0.011	0.081; 0.012
Male	16.8 ± 19	16.7 ± 17			
Female	11.3 ± 14	16.1 ± 20			
**Ice Cream**			0.23; 0.006	<0.001; 0.11	0.90; <0.001
Male	9.5 ± 12	3.3 ± 3			
Female	11.0 ± 15	5.0 ± 6			
**Candy**			0.84; <0.001	0.58; 0.001	0.92; <0.001
Male	7.5 ± 10	5.9 ± 7			
Female	6.9 ± 9	5.5 ± 6			
**Sauces** ^**c**^			<0.001; 0.066	0.65; 0.001	0.27; 0.005
Male	16.7 ± 12	18.3 ± 19			
Female	11.2 ± 11	10.5 ± 11			
**Salt (added)**			0.46; 0.002	0.25; 0.005	0.95; <0.001
Male	0.23 ± 0.4	0.17 ± 0.3			
Female	0.27 ± 0.5	0.25 ± 0.5			
**Fruit Juice**			0.64; 0.001	0.91; <0.001	0.23; 0.006
Male	140.0 ± 138	159.6 ± 157			
Female	168.5 ± 198	152.2 ± 156			
**Energy Drinks**			0.065; 0.013	0.27; 0.005	0.003; 0.035
Male	5.9 ± 17	2.7 ± 6			
Female	1.3 ± 4.3	2.9 ± 10			
**Soft Drinks (pop/soda)**			0.91; <0.001	0.60; 0.001	0.30; 0.004
Male	51.8 ± 70	65.0 ± 97			
Female	58.5 ± 130	54.2 ± 140			
**Beer**			<0.001; 0.18	<0.001; 0.055	0.015; 0.023
Male	108.3 ± 145	146.4 ± 206			
Female	23.8 ± 59	31.7 ± 82			
**Wine **			0.087; 0.011	0.22; 0.006	0.076; 0.012
Male	6.8 ± 23	5.7 ± 15			
Female	7.9 ± 21	14.3 ± 25			
**Liquor**			0.14; 0.008	<0.001; 0.10	0.002; 0.036
Male	29.9 ± 50	75.7 ± 106			
Female	33.1 ± 66	45.5 ± 61			

All results are shown as mean ± SD

* Significance from 2-way RMANOVA (Group: sex; Time: beginning to end), significantly different with P value ≤0.05, and effect size was determined by partial eta-squared.

^a^ Muffins, scones, sweet rolls, danishes, pop tarts, pancakes, waffles

^b^ Burgers, hot dogs, sausage/bacon, ribs, tacos, pork, lunch meat, mixed meat dishes

^c^ BBQ sauce, mayonaise, ketchup, gravy

Donuts/cakes, macaroni and cheese, salt with vigorous physical activity as a covariate; Donuts/cakes with living arrangement as a covariate; candy, beer with facutly as a covariate.

Table 4 depicts foods that are generally seen as unhealthy along with their accompanying intakes in g/day for males and females. Significant time effects indicating reductions in intake for both sexes were observed for pastries, fried fish, meat dishes and ice cream. Time effects indicating increased intakes for both sexes were observed for fried chicken, beer and liquor. Significant interactions indicated that donuts/pastries, fried chicken, beer and liquor increased more in males, and energy drinks increased more in females. French fries and wine tended to increase more in females than males. Intakes of pizza, macaroni and cheese, candy, sauces, added salt, fruit juice, energy drinks, soft drinks, and wine did not significantly change from beginning to end of the study.

### Correlations

There were several significant (all p<0.05) and plausible correlations between changes in dietary variables and changes in body composition variables, and/or changes within 2 dietary variables. These were all exploratory in nature. For example, change in waist circumference was positively correlated with changes in saturated fat (R = 0.37) and sodium intake (R = 0.36) as well as pizza (R = 0.39) and fried chicken (R = 0.36), and negatively correlated with changes in yogurt intake (R = -0.43), but only in males that gained body weight (n = 46, mean weight gain = 4.3 ± 3.1 kg). Likewise, change in fat mass negatively correlated with change in yogurt (R = -0.37) and positively correlated with changes in fried chicken (R = 0.53) in males that gained body weight. For females that gained body weight (n = 155; mean weight gain = 2.8 ± 2.5 kg), change in waist circumference was negatively correlated with carrot intake (R = -0.28) and positively correlated with alcohol intake as mg of ethanol (R = 0.16) and a composite term for all alcoholic beverages (beer, wine and liquor combined; R = 0.18). Apple (R = -0.15), carrot (R = -0.27) and liquor intake (R = 0.16) were significantly correlated with waist circumference in all females (n = 210) but not males. In terms of fat mass or %body fat, oranges (R = -0.17), broccoli (R = -0.14), tomatoes (R = -0.14), corn (R = -0.15) and energy drinks (R = 0.14) were significantly correlated in all females but not males; whereas temperate fruit (R = -0.28), yogurt (R = -0.38), cheese (R = -0.31) and fried chicken (R = 0.45) were significantly correlated in all males (n = 50) but not females. In terms of correlated foods/beverages, juice intake was significantly positively correlated with total calories (all males: R = 0.33; all females: R = 0.20) and total sugars (all males: R = 0.57; all females: R = 0.52), as was soft drinks with total sugars (all males: R = 0.29; all females: R = 0.26). Beer intake was significantly positively correlated with liquor intake (R = 0.34) in all males and wine intake (R = 0.23) in all females.

## Discussion

This study provides important insight regarding the sex-specific changes in body weight and adiposity, and sheds light on some sex-specific changes in dietary intake and quality in first year university students. There were 4 main findings from this study. 1) Males and females display different patterns of body weight and body composition changes during first-year university (Table 1). Both males and females gain a significant amount of body weight (and BMI) with males gaining more than females. Males also had greater absolute increases in waist circumference, waist to hip ratio, fat mass and lean mass than females, but when fat mass was expressed as a percent of total weight gained, females gained more fat than males. 2) Total energy intake remained the same throughout the year, with no difference between sexes. This was accompanied by no change in most nutrients except for an increase in alcohol (mg ethanol) in both sexes but more so in males (Table 2). 3) Diet quality decreased over time. Specifically, overall patterns of intake show that the consumption of most healthy foods/beverages decreased, a few remained the same and none increased (except for coffee; Table 3), whereas the consumption of unhealthy foods/beverages either increased, decreased or remained the same (Table 4). These adverse changes were more pronounced in males versus females. For example, males displayed greater reductions in vegetable intake and greater increases in donuts/cakes, fried chicken, beer and liquor compared to females. This indicates a decrease in overall diet quality and an adoption of unhealthy eating habits. 4) Some dietary intake changes correlated with fat mass and waist circumference change indicating that poor dietary choices were associated with increased adiposity. Thus, our research demonstrates that sex differences do exist in several of the measured variables indicating that males and females respond differently in terms of diet and body composition/anthropometry change during this critical time. Indeed, males demonstrated greater overall adverse changes.

In the media and on campuses, weight gain is commonly referred to as the “Freshman 15” [5, 23], which is the notion that students in their first year of university gain, on average, 15 lbs. While this is a popular general concept, it is not supported by most research [5, 23]. A meta-analysis from 2009 of 24 studies with a total of 3,401 participants (85% female) calculated that the average weight gain was only 1.8 kg (4.0 lbs) during first-year university [5]. A more recent and updated meta-analysis (2015) of 22 longitudinal studies (5,549 students) calculated an average weight gain of only 1.4 kg (3.1 lbs) during first-year university [23]. Within these meta-analyses, there were wide ranges of weight change; 0.7 kg to 4 kg (1.5 lbs to 8.8 lbs) [5] and -0.7 kg to 3.1 kg (-1.5 lbs to 6.8 lbs) [23]. Despite average weight changes generally being of smaller magnitude, they still represent a weight gain in these students that is 5 times greater than the general population over a year [49]. In addition, some studies in which both males and females are studied, show that males typically gain more weight than females [3, 5, 13, 14]. Taken together, these meta-analyses demonstrate (in ~9000 students) that an average modest weight gain does occur, however, they provide no information on the composition of the weight gained and little discussion on the factors that may lead to this weight gain. Our results are consistent with these general patterns and are on the higher end of the reported ranges demonstrating body weight gains of 3.8 kg (8.4 lbs) in males and 1.8 kg (4.0 lbs) in females, and body fat gains of 2.7 kg (6.0 lbs) and 1.5 kg (3.3 lbs), respectively. While these gains are less than the alleged 15 lbs, research has documented that even a 1 kg gain in body weight (or a 1 cm gain in waist circumference) is associated with increased disease risk [50, 51].

Trends in weight gain during this period are well documented, however, the assessment of body composition is less so. The latter is important because it provides insight into healthy (i.e. increases in lean mass) or unhealthy (i.e. increases in fat mass) weight change [50, 52]. We demonstrated that while males and females both gained weight, males gained more absolute weight in all compartments compared to females, which was likely reflective of their greater total weight gain. However, females gained relatively more fat mass and relatively less lean mass than males. This difference is not necessarily able to be explained by divergent dietary intakes and may relate to other lifestyle factors like physical activity. Indeed, and published elsewhere [47], all intensities of physical activity (light, moderate, vigorous) significantly decreased in both sexes over the year. However, despite an overall reduction in physical activity minutes, our research also demonstrated an increase in “other [physical] activities” over the year, and males cited their other activity of choice as weight lifting/resistance training (whereas females cited fitness classes). Although overall physical activity decreased, this may have impacted our lean mass results. Nevertheless, fat mass and waist to hip ratio increased in both groups, and more so in males.

Energy intake decreased by 250–400 kcal/d over the year (Table 2). This decrease, while significant, is accounted for by participants’ living arrangements (covariate). A reduction in total energy intake accompanied by an increase in body weight was previously observed by Butler et al., in 54 female first-year students [29] and by Jung et al., in 66 female first year students [31]. Butler et al. [29] used a FFQ, and Jung et al. [31] used 3-day diet records. Reasons for decreased energy and food/nutrient intake that are not congruent with body weight/composition results may relate to various factors including underreporting [45, 53], a greater awareness of food consumption, the new need for students to shop for, cook and prepare their own food, and the general higher cost of food, particularly healthy food [24, 27, 54]. Given that most students in our study were living in residence and on a meal plan (and therefore did not need to cook/buy their own food per se), it is possible that other factors are contributing to a decreased energy intake including a lack of a variety of foods. Future research should continue to explore these barriers to healthy eating in this population possibly also under different living situations.

Many studies that report modest dietary intake changes (positive or negative) over the academic year have found no significant associations between diet and weight or body composition change [28, 55]. This indicates that long-term changes in recorded (and usually self-reported) dietary intake may not be the main driver (or may only be part of the puzzle) of overall weight change in this population. However, using an exploratory approach, our study did find significant correlations between fat mass and waist circumference with nutrient/food intake changes indicating a relationship between the changes in dietary intake and body composition/adiposity. For example, saturated fat and sodium intake as well as pizza and fried chicken were negatively correlated with body composition changes in males who gained weight. Additionally, fruit, yogurt and cheese intakes were negatively correlated with fat mass in males.

Our study demonstrated negative changes in diet quality for both sexes, supporting previous research in young adults, particularly those in university, citing decreases in diet quality such as frequent fast food consumption, decreased dairy, fruit and vegetable intakes, decreased consumption of whole grain carbohydrates and increases in energy drinks, alcohol and pop/soda [4, 36, 56–66]. A decrease in diet quality may also be explained by the fact that students are living away from home, they are solely responsible for feeding themselves, there are more social pressures to eat or not eat certain foods/beverages [67] accompanied by less parental influence [68] and lack of self-control [27, 69], a greater availability and lower cost of poor quality foods [70], and a general lack of nutrition knowledge [71, 72]. Again, given the fact that most of our participants lived in residence, another factor could be the types of foods that are available on campus [73, 74]. Although there is provision of a variety of foods on our campus, food options which are convenient, fast and readily available for lower prices tend to not be as healthy. This trend is wide-spread across post-secondary institutions [74, 75]. A novel finding from our study is the divergent eating patterns of males versus females. While both sexes demonstrated shifts towards poorer diet quality, males made larger negative dietary changes than females.

An interesting finding from this study pertained to alcohol and other beverage intakes. Alcohol (mg of ethanol/day) and consumption of alcoholic drinks (beer and liquor) significantly increased. In addition, soft drinks (pop/soda), energy drinks and fruit juice intake did not significantly change over time, however females had a greater increase in energy drink consumption than males. Thus, by the end of the study, alcohol and potentially other sugary/caffeinated drinks represented a higher percentage of total energy intake. This may help to partially explain the increase in body weight, waist circumference and fat mass observed in both sexes. Several studies in first-year cohorts have shown similar results [28, 29, 76]. In particular, Butler et al., showed significant increases in alcohol intake in female students both as a % of total energy intake and as the number of beverages per day, while also demonstrating a significant weight gain [29]. Our study extends these findings to a larger sample size and particularly, to males. Also, in accordance with the literature, males tended to have a higher consumption of alcohol compared to females [66, 77]. The greater increase in alcohol may relate to the greater waist circumference, waist to hip ratio and fat mass observed in males versus females, as excess calories from alcohol are more likely to be deposited as abdominal fat rather than fat elsewhere in the body [78]. This is concerning since increased abdominal fat is preferentially associated with an increased risk of chronic disease (diabetes and heart disease) [52, 77, 79].

Strengths of our study included the longitudinal design, measured rather than self-reported body weight, a larger sample size, as well as the measurement of a comprehensive set of variables including anthropometry, body composition and nutrient/food intake. Furthermore, the inclusion of both males and females in our sample allowed for direct comparisons of all variables between the sexes, enabling us to uncover interesting findings relating to differential changes during this critical time. Only a few studies have directly compared between sexes and only for some of these variables [13, 80, 81]. Most research in first-year students has focused on females and did not measure body composition [5, 23].

Limitations of our study may relate to the use of the FFQ since the possibility of inaccurate estimation of dietary intake may have affected our results. Underreporting or misreporting seems to be a general issue when assessing habitual diets over the long term [45]. This may be due to a lack of precision of the instrument (i.e. the FFQ), the inability of the individual to accurately estimate and report their long-term intake, and/or the reluctance of the individual to truthfully report intake. This phenomenon of nutritional misreporting has been widely cited in the literature [53, 82–84], and can be an issue with all methods of self-reported dietary assessment. While we are aware of this issue, misreporting should affect the results at both time points, and therefore cannot entirely explain the observed differences over the academic year or between sexes. Moreover, validated self-reporting tools are still the most widely used methods for nutrition data collection [19]. Another limitation may relate to our classification of healthy vs. unhealthy foods. Although our categorization is based on resources like Canada’s Food Guide [40] and related published meta-analyses [43, 44], some foods may be misclassified for some people based on *how* they were consumed. For example, eating too much read meat is not advised, neither is too much pasta or pasta with a high-fat sauce. Nonetheless, to account for some of these factors, we separated healthy vs. unhealthy versions of foods when this information was available from the FFQ. For example, pasta was labeled as healthy, but macaroni and cheese was labeled as unhealthy. Steak and poultry were labeled as healthy, whereas meat dishes (hot dogs, hamburgers, lunch meat, etc.) and fried chicken were labeled as unhealthy. Another limitation may be that we focused our study on diet-related changes and did not discuss changes in habitual physical activity. Physical activity data from this sample has been published elsewhere [47]. Decreases in the amount (i.e. energy expenditure) and changes in the type (i.e. aerobic vs. resistance exercise) of physical activity could further explain the increase in body weight, fat and lean mass, as well as the different ratios of fat:lean mass between the sexes. To help account for these potential effects, 3 levels of physical activity (light, moderate and vigorous) were assessed as covariates and if significant, were included in the individual RMANOVA models for the anthropometric, body composition and nutritional intake data. Lastly, our study may have incurred an element of sampling bias in that potentially healthier students who are curious about their eating habits and body changes over the year were more inclined to participate. If this was indeed the case, our changes would have potentially been greater than what we observed if less ‘health-minded’ students participated.

## Conclusions

Results from our study demonstrate that young male and female students undergo unfavorable and differential changes to their dietary intakes during the transition to university life. These changes reflect a poorer quality diet for both sexes, but more so for males, and were accompanied by increases in body weight, BMI, waist to hip ratio and body fat, which could lead to possible longer-term health implications and increased disease risk [50, 51]. Sex-specific changes were evident for nutrition indicating that males’ diets were lower in quality, and body composition changes indicated that males experienced more adverse changes by gaining more body weight, waist circumference and fat mass than females. Future research should focus on mitigating these adverse changes particularly during times of stress through the development of effective sex-specific interventions targeted at improving dietary habits and nutritional knowledge, as well as mitigating adverse body composition changes as students transition into university life.
